# Cancer of the Prostate

**Published:** 1903-11-14

**Authors:** 


### CANCER OF THE PROSTATE.

Malignant disease of the prostate is probably regarded by the majority of surgeons as a condition which does not require much consideration from the standpoint of operative practice except in so far as palliative measures may be required for the relief of pain or some incidental complication. Dr. R. H. Greene[1] is of opinion that, in the light of recent inquiries, this is not the correct position for the surgeon to take. He believes it to be possible in some instances to diagnose or to have sufficiently strong suspicions of the presence of prostatic cancer to warrant active measures at a stage when the disease is purely local, and amenable therefore to a radical operation with the chance of permanent cure. He believes also that much may be done in the way of prophylaxis.

The frequency of the condition is difficult to determine. Granted that the so-called hypertrophied prostate of the aged is a chronic inflammatory process, as now seems evident, it is natural to expect, Dr. Greene argues, that cancer will follow in quite a large proportion of such cases, as it follows chronic inflammatory conditions occurring in other portions of the body. Indeed, he estimates that cancer occurs in a proportion as high as 5 to 10 per cent. of old men suffering from prostatic diseases. In order to obtain a correct estimate it would be necessary to examine a large number of prostates and to make many sections through each; otherwise a cancer occurring in one lobe might be overlooked. A practical point of considerable importance is the early infection of the lymphatic glands which occurs in cases of cancer in this region. If a radical operation is to be done the true condition must be recognised and the operation carried out before the lymphatic glands have been affected.

The early diagnosis must be made by inference. The patient will usually be past middle life, and there will generally be a history of symptoms pointing toward trouble with the prostate extending over several years. Cachexia may be of diagnostic significance if it comes on comparatively suddenly, without other obvious reasons to account for it, in a patient who has an enlarged prostate. The most frequent symptom of all is pain, and this may be the first thing to awaken in the observer a suspicion of malignant disease. The pain may be referred to the prostate, that is to the perineal region, to the rectum, to the back, over the region of the kidney, to the bladder, or glans penis, or to the distribution of peripheral nerves, *e.g.* branches of the sciatic. As the pain may occur before metastasis into the neighbouring lymphatic glands has taken place, its presence in an old prostatic case should lead to early surgical intervention, unless the reasons against it are of the strongest. Hæmorrhage ranks next to pain in point of frequency as a symptom of prostatic cancer. It occurs in about 50 per cent. of all cases. Careful observation with the cystoscope may determine the source of the bleeding in such an instance. Physical examination may yield no useful information; on the other hand, the sudden increase in size of a prostate as determined by a rectal or urethral examination, is often indicative of malignant disease.

In discussing the treatment Dr. Greene considers it under three heads, namely, preventive, palliative, and curative. He remarks that "the ultimate analysis of the question of preventive treatment seems to lead to the conclusion that if gonorrhœal infection could be prevented in the first place, the occurrence of cancer of the prostate would become much less frequent; for it is now well established that chronic posterior urethritis is a very frequent complication of acute urethritis. More and more evidence is being brought out to demonstrate that chronic posterior urethritis and chronic prostatitis go hand in hand, that the latter plays a causative part in the formation of the so called prostatic hypertrophy, and that this in turn, reasoning from analogy, plays a part in the frequency of the formation of cancer." He adds, also, that even when gonorrhœa has already occurred much may be done by a more prolonged and careful treatment of chronic posterior urethritis and prostatitis than is usually carried out, towards preventing the prostatic enlargement and the tendency to secondary cancer. Palliative measures may be undertaken with a view to prolonging life and making it more bearable, but much thoughtfulness and care are required before advising such a procedure as the making of a permanent suprapubic opening or a partial operation on the prostate itself, as instances have been recorded where such procedures have failed in their object, and, indeed, have seemed to enhance the patient's suffering. Curative treatment consists of complete removal of the cancerous prostate before metastasis has taken place.

[1] New York Medical Journal, October 24.

